# Effective Thermal Expansion Property of Consolidated Granular Materials

**DOI:** 10.3390/ma10111289

**Published:** 2017-11-09

**Authors:** Gülşad Küçük, Marcial Gonzalez, Alberto M. Cuitiño

**Affiliations:** 1 Department of Mechanical and Aerospace Engineering, Rutgers University, Piscataway, NJ 08854, USA; gulsad@gmail.com; 2 School of Mechanical Engineering, Purdue University, 585 Purdue Mall, West Lafayette, IN 47907, USA; marcial-gonzalez@purdue.edu

**Keywords:** effective thermal expansion coefficient, thermo-mechanical coupling, granular materials, particle mechanics, contact mechanics, thermally-assisted compaction

## Abstract

Thermally-assisted compaction of granular materials is of keen interest in many engineering applications. A proper estimation of the material behavior of compacted granular materials is contingent upon the knowledge of microstructure formation, which is highly dependent on the bulk material properties and processing conditions, during the deformation stage. Originating from the pair interactions between particles, the macroscopic properties are obtained using various homogenization techniques and postulating continuum constitutive laws. While pioneers in this field have laid fundamental groundwork regarding effective medium descriptions, there exists a discrepancy between discrete and continuum level solutions. In our previous work, we elaborated a Particle Mechanics Approach (PMA) that integrates thermal contact and Hertzian deformation models to understand the thermo-mechanically-coupled consolidation problem. We also considered the analogous problem from the perspective of the conventional Continuum Mechanics Approach (CMA). In this study, following the multi-scale modeling framework, we propose an effective thermal expansion coefficient for the thermally-assisted compaction of granular materials.

## 1. Introduction

In-depth understanding of the thermo-mechanical coupling that takes place during thermally-assisted compaction of granular materials is the key to successful design and optimization of many powder process engineering applications. It is well known that the elastic properties of bulk materials are altered by a change in temperature. Moreover, as also pointed out by Jaeger et al. [1], granular materials exhibit exceptional properties, which differ from those of the primary bulk material and depend on the processing conditions of the granular system. In this regard, there is a significant number of efforts to elucidate the collective behavior, which is also referred to as effective thermo-mechanical properties, of granular materials in response to boundary conditions and system parameters.

Mathematical models were developed to understand the thermal contact of conforming elastic surfaces [2,3,4]. Starting from the definition of thermal flux at the smooth contact surface of two spherical particles, Batchelor and O’Brien solved the analytical equation that expresses the effective thermal conductivity of ordered and randomly-distributed packed granular assemblies [4]. Experimental studies validating these models were presented in the related work of Hadley [5], Nozad et al. [6] and Shonnard and Whitaker [7]. More refined studies relaxed these assumptions by focusing on elasto-plastic contacts [8] or rough non-conforming surfaces [9,10,11]. Recently, the field of porous media has continued to attract researchers in light of understanding the correlation between geometry, loading conditions and anisotropy in the microstructure, which in turn affect the macroscopic behavior of compacted beds [12]. Yun and Santamarina conducted a fundamental study on thermal conduction through one-dimensional granular chains of spherical particles [13]. They pointed out the decisive role of inter-particle contacts in heat transfer mechanisms and highlighted the need for the effective thermal conductivity models to consider the inherent presence of contacts in particulate materials [13]. Exploring the effects of packing grains during thermal cycling, Chen and his co-workers [14] showed that thermal expansion, due to an imposed thermal gradient, has a significant effect on the rearrangement of the particle bed. Moreover, heat transfer in granular flows has been of great interest for numerous industrial applications, particularly in regards to understanding the effect of process conditions on heat transfer mechanisms in granular media [15,16,17,18,19].

In recognition of the uniqueness of granular materials and their importance to a wide variety of thermally-assisted compaction processes, two main methodologies have received considerable attention for addressing the need for simulating and predicting the macroscopic behavior of granular materials. One of the most adopted approaches treats the particles as individual bodies such that the coupled effects of various multi-physical phenomena are described at the particle-scale. Accounting for particle interactions and adopting constitutive relations of contact mechanics [20,21,22], the discrete element approach has been widely used in the field of particle-scale research [23]. This method is based on the early work of Cundall and Strack, where authors introduced an explicit numerical scheme to describe granular dynamics by tracing the motion of the particles and the generation of forces over the contact network while solving discrete equations of motion [24]. The interrelationship between particle motion and energy with the macroscopic behavior of the assembly provides understanding of the overall behavior of the confined material [25]. The main advantage of this methodology is the capability of presenting broad information about the microstructure of the granular material [26]. Although there exists a considerable amount of computational challenges in modeling a large number of particles with discrete element methods, improvements were done in formalisms, and new simulation techniques increased the achievability of calculations at the particle level.

The second most implemented methodology to model the collective behavior of particulate materials is the continuum mechanics approach, in which the granular material is assumed to be statistically homogeneous [27]. This simplification is achieved by treating the system as units of ordered arrays, simulating disordered arrangements by statistical correlation functions or using empirical correlations. The statistical averaging techniques provide homogenized solutions of the highly heterogeneous granular media at the cost of imposing two assumptions: (i) affine motion approximation, namely the motion of each grain follows the macroscopic strain, and (ii) well-bonded structure, contact number and positioning do not change under the applied load. Despite the fact that effective medium theory particularly estimates the effective elastic moduli of a packed bed of spherical particles to a large extent, the discrepancy between numerical and experimental results is remarkable. Makse and co-workers questioned the relevance of force laws defined at the single contact level, where they pointed out that the simplification done in effective medium theory is the misleading element in the formulation [28,29]. The affine motion assumption demolishes the ability of the approach to account for the relaxation and rearrangement of particles that are under shear deformation. Moreover, concerning the variety of boundary conditions and geometrical effects, experimentation techniques become insufficient in providing reliable information about the microstructure to feed empirical correlations.

The improvement of theoretical models and numerical simulation schemes remains an active area of research in the study of granular matter. A quasi-continuum approach has been recently developed, and the formulation was used for simulation of inter-particle bonding in granular systems [30]. Zheng and Cuitiño implemented the quasi-continuum approach to bridge the micro- and meso-scale through a discrete-continuum formulation of elastic-inelastic deformations occurring in the post-rearrangement regime of consolidation of inhomogeneous granular beds [30]. Since this approach provides the flexibility of storing individual particle interactions in an finite element modeling (FEM) scheme, it provides the overall behavior of the entire body without losing critical information specific to the microstructure. Koynov et al. presented a notable adaptation of this approach on the topic of powder compactions for pharmaceutical purposes [31]. Gonzalez and Cuitiño introduced a new formulation that accounts for the interplay of nonlocal mesoscopic deformations characteristic of confined granular systems. In the absence of the classical restriction of independent contacts of the Hertz law, the extended theory of nonlocal contact formulation provides predictive models at moderate levels of deformation and high confinement [32].

The present work attempts to establish a relationship that defines the effective thermal expansion property of a packed bed of spherical particles under the effect of the thermally-assisted compaction process. We elaborate on a Particle Mechanics Approach (PMA) that entails the integration of contact mechanics principles with a thermal-contact model to account for the heat conduction at the quasi-static equilibrium of the deformed state of granular materials. The disordered nature of the problem leads to highly non-linear coupled equations; therefore, we investigate a regular packing to simplify the problem and make it mathematically traceable. Moreover, we consider the analogous problem from the perspective of the conventional Continuum Mechanics Approach (CMA), while practicing the effective medium approach descriptions that define the macroscopic thermal and mechanical properties. Similar in spirit to the work of Chan and Tien [2], who proposed the effective thermal resistance, and to the work of Walton [33], who presented a method to calculate the effective elastic moduli of granular packing, we adopt a multi-scale approach to link the particle level information to the continuum level description of the thermally-assisted compaction process. Finally, we derive the equation of effective thermal expansion coefficient for the regularly-packed bed of particles.

## 2. Mathematical Models

### 2.1. Particle Mechanics Approach

Particle scale modeling of the thermally-assisted compaction process requires an extension of the discrete element method to account for the integration of heat conduction (e.g., [25,34,35]). Starting from the well-known theory of Hertzian deformation [20], heat conduction through the conforming contact of spherical particles [2,4] is adopted for the case of thermally-assisted compaction. Under steady state conditions, the total of the forces acting on individual particle *m* from neighboring particles $n\in N_{m}$ and the total heat transferred to particle *m* are zero, that is:

(1) $$F^{m}=\sum_{n\in N_{m}}F^{mn}n^{mn}=0$$

(2) $$Q^{m}=\sum_{n\in N_{m}}Q^{mn}=0$$

where $n^{mn}$ is the unit normal vector defined from $x^{n}$ to $x^{m}$, i.e., from the center of particle *n* to the center of particle *m*.

Johnson studied the elastic deformation of locally spherical particles that are subject to a compression load by contact mechanics considerations [36]. Small-strain deformation of conforming surfaces results in a flat circular contact area. The collinear, elastic, contact force between particles *m* and *n* is defined through reduced elastic modulus $E^{mn}$, reduced particle radius $R^{mn}$ and overlap $\gamma^{mn}$ between these particles. Specifically, the contact force is:

(3) $$F^{mn}=\frac{4}{3}E^{mn}{\left(R^{mn}\right)}^{1/2}{\left(\gamma^{mn}\right)}^{3/2}$$

where:

(4) $$\begin{array}{ccc} R^{mn} & = & {\left[\frac{1}{R^{m}}+\frac{1}{R^{n}}\right]}^{-1} \end{array}$$

(5) $$\begin{array}{ccc} E^{mn} & = & {\left[\frac{1-{\left(\nu^{m}\right)}^{2}}{E_{m}}+\frac{1-{\left(\nu^{n}\right)}^{2}}{E_{n}}\right]}^{-1} \end{array}$$

(6) $$\begin{array}{ccc} \gamma^{mn} & = & R^{m}+R^{n}-∥x^{m}-x^{n}∥ \end{array}$$

Similar to previous studies in the literature [35,37], in the present study, a linear thermal expansion formulation is taken into consideration; that is $R^{m}=R_{ref}^{m}\left[1+\alpha^{m}\left(T^{m}-T_{ref}^{m}\right)\right]$, where $\alpha^{m}$ is the thermal expansion coefficient, $T_{ref}$ is the reference temperature and $R_{ref}^{m}$ is the radius of the particle at the reference temperature. Due to the fact that the contact geometry depends highly on the heat conduction between the consecutive conforming particle pairs, it is expected to capture a distribution of the contact area formation throughout the compacted medium.

The major heat transfer mechanisms in compacted particle beds consist of conduction through solid particles, conduction through the contact area between two touching particles, conduction to/from interstitial fluid, heat transfer via convection, radiation between particle surfaces and radiation between neighboring voids [34]. For a system of granular media where the thermal conductivity of the solid particles is much larger than that of the interstitial medium, the driving mechanisms for the heat transfer are the first two. Restricting attention to the problem of thermally-assisted compaction of spherical particles in a vacuum, we focus on thermal contact models that consider the conduction through solid particles and the contact areas between touching particles.

The analytical solution of the heat conduction through the solid phase of ordered spherical particles has been proposed by Chan and Tien [2] and Kaganer [3]. Moreover, the problem of heat transfer regarding the compaction of particles that are in or nearly in contact is deeply investigated by Batchelor and O’Brien [4]. In an attempt to find the approximate effective thermal conductivity of ordered and randomly-packed granular beds, Batchelor and O’Brien discussed the heat flux across the flat, circular contact surface between smooth, conforming and elastic particles. In this study, we adopt Batchelor and O’Brien’s model for predicting the heat conductance, which is the ability of two touching surfaces to transmit heat through their contact interface. Heat flux across the contact area of two spherical, smooth particles is given by:

(7) $$Q^{mn}=2a^{mn}k^{mn}\left(T^{m}-T^{n}\right)$$

where $k^{mn}$ is the arithmetic mean of the thermal conductivities of two conforming particles and $a^{mn}$ is the Hertzian contact area. These are defined as:

(8) $$\begin{array}{ccc} k^{mn} & = & \frac{1}{2}{\left[\frac{1}{k^{m}}+\frac{1}{k^{n}}\right]}^{-1} \end{array}$$

(9) $$\begin{array}{ccc} a^{mn} & = & \sqrt{\gamma^{mn}R^{mn}} \end{array}$$

The total heat flow to an individual particle, Equation (2), is calculated by adding the heat flow, Equation (7), across each contact surface shared with its neighboring particles. Thermal contact models introduced in the literature [2,4], Equation (2), assume that the resistance to heat transfer inside the particle is significantly smaller than the resistance between the particles, i.e., a Biot number much less than one:

(10) $$\begin{array}{ccc} \frac{2k^{mn}a^{mn}}{k^{mn}A/R^{m}} & \ll & 1 \end{array}$$

where *A* is the cross-sectional area, $A=\pi {\left(R^{m}\right)}^{2}$. This assumption was applied by several authors in earlier studies [34,38], which also enforces the condition of $a^{mn}\ll R^{m}$, i.e., of small-strain deformation of elastic bodies in contact.

**Figure 1:**
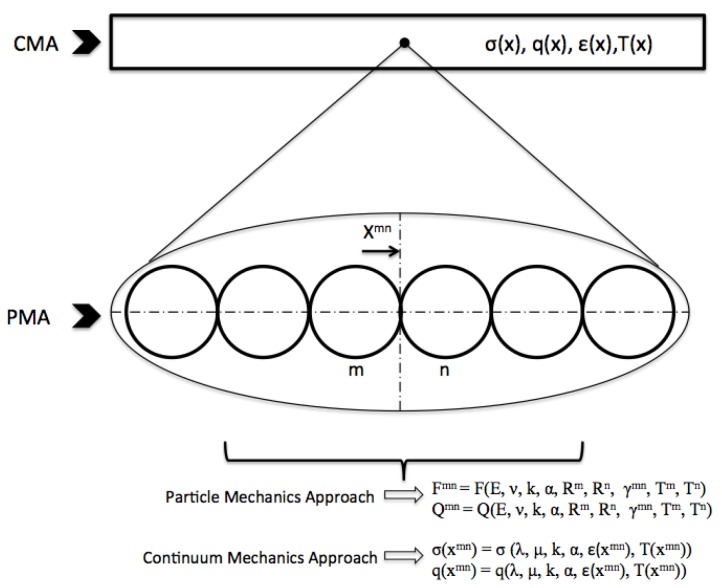
Particle and continuum mechanics approaches.

Referring to the previous experimental studies on regular and random packing of granular media, Walton points out that although the regular packing models are founded on strict assumptions, they are capable of capturing the vast majority of the characteristics of a real granular media [33]. In the present study, we consider a simple cubic packing of identical elastic spheres, which are constrained between parallel planes of infinite extent. A compression load and a temperature gradient are applied along the major and finite direction. Stress and heat flux are defined to depend only on externally-applied thermal and mechanical loads, and the weight of the particles is neglected. For such regular packings, each layer of the arrangement is isothermal normal to the direction of applied load. Furthermore, since these transversely-oriented particles are, at most, at the contact point, for each particle there is only one pair of contact areas aligned with the direction of applied thermal and mechanical load. Due to the symmetry of the problem, it is sufficient to consider a single column of a square cross-section containing the longitudinally-compressed spheres together. The above-described set of concepts regarding regular packings is also encountered in the early work of Chan and Tien [2] and Kaganer [3]. Based on these assumptions, the specified granular media can be visualized as a chain of elastic particles compressed between two walls, which are maintained at different temperatures, as seen in Figure 1. Details of the particle mechanics approach adopted in this study can also be found in detail in our earlier work [39].

### 2.2. Conventional Continuum Mechanics Approach

There has been considerable research directed towards describing the macroscopic behavior of compacted granular materials by using various homogenization techniques and postulating continuum constitutive laws [40]. Some of the previous studies on mathematical modeling of transport properties are aimed at estimating elastic-plastic mechanical properties, thermal and electrical conductivity of ordered and disordered arrangements. In addition to the particle-level approach, we also focus on a small-strain thermoelasticity model of continuum scale description that integrates the previously-proposed effective mechanical and thermal properties for granular beds under compaction. In this study, we refer to the particle mechanics approach and the conventional continuum mechanics approach as PMA and CMA, respectively.

The governing field equations of motion and energy of the analogous problem defined at the continuum scale are the following: $\mathrm{div}\left(\sigma \right)=0$ and $\mathrm{div}\left[k\;\mathrm{grad}\left(T\right)\right]=0$, where Cauchy’s stress, σ, is formulated as a combination of classical linear elasticity theory and simple linear thermal expansion, that is:

(11) $$\begin{array}{ccc} \sigma & = & \lambda \mathrm{tr}\left(\varepsilon \right)I+2\mu \varepsilon -\left(3\lambda +2\mu \right)\alpha \left(T-T_{ref}\right)I \end{array}$$

where I is the identity matrix. The solution for the basic one-dimensional steady state thermoelastic, continuum problem, where body forces are neglected, depends linearly on elastic constants, λ, μ, thermal expansion and conduction coefficients, α and *k*, respectively. Since $\varepsilon_{22}=\varepsilon_{33}=0$ holds, $\varepsilon_{11}$ is referred as ε(x), and it is defined positive for compression. The system of questions then reduces to:

$$\sigma \left(x\right)=\left(\lambda +\mu \right)\varepsilon \left(x\right)+\alpha \left(3\lambda +2\mu \right)(T\left(x\right)-T_{ref}),$$

with σ(x) positive for compression, and q=k∂T/∂x.

Effective mechanical properties of granular beds are of great interest for numerous theoretical studies, some of which focuses on: (a) the principal elastic modulus for vertical compression of spherical particles without any lateral extension (Walton [33]); (b) finite and incremental elasticity of random packing of identical particles using energy methods (Norris and Johnson [41]); (c) enhancement of the derived formulas based on the pressure dependence of the elastic moduli of granular packings (Makse et al. [28,29]). The effective medium approach proposes the following elastic effective properties, $\tilde{\lambda }$ and $\tilde{\mu }$:

(12) $$\begin{array}{ccc} C_{n}=4\frac{\mu }{1-\nu } & = & 4\frac{E}{2(1+\nu )}\frac{1}{1-\nu }=\frac{2E}{1-\nu^{2}} \end{array}$$

(13) $$\begin{array}{ccc} \tilde{\lambda }+2\tilde{\mu } & = & \frac{3}{20\pi }C_{n}{\left(\phi_{s}Z\right)}^{2/3}{\left(\frac{6\pi \sigma }{C_{n}}\right)}^{1/3} \end{array}$$

(14) $$\begin{array}{ccc} 3\tilde{\lambda }+2\tilde{\mu } & = & \frac{1}{4\pi }C_{n}{\left(\phi_{s}Z\right)}^{2/3}{\left(\frac{6\pi \sigma }{C_{n}}\right)}^{1/3} \end{array}$$

where $C_{n}$ is the actual stiffness that depends on the bulk mechanical properties: Young’s modulus, *E*, and Poisson’s ratio, ν. $\phi_{s}$ is the packing fraction, and *Z* is the coordination number.

The effective thermal conductivity of a granular bed is substantially sensitive to the thermal and elastic properties of individual particles. In this study, we adopt Batchelor and O’Brien’s [4] solution for effective thermal conductivity coefficient:

(15) $$\begin{array}{ccc} \tilde{k} & = & k{\left(\frac{6\sigma }{C_{n}}\right)}^{1/3} \end{array}$$

It has been shown that the above-mentioned thermal contact models provide accurate results in estimating steady and average temperature profiles for ordered granular packings [42].

After implementing the effective mechanical and thermal properties in the resembling continuum description, the equation of stress becomes:

(16) $$\begin{array}{ccc} \sigma & = & \phi_{s}ZC_{n}{\left(\frac{3}{32\pi^{2}}\right)}^{1/2}{\left[\varepsilon \frac{3}{5}+\alpha \left(\frac{T_{2}^{w}+T_{1}^{w}}{2}-T_{ref}\right)\right]}_{+}^{3/2} \end{array}$$

where $T_{1}^{w}$ and $T_{2}^{w}$ are the temperature at the constraining walls, and ε is the compaction strain along the principle direction. The overall compaction force can simply be expressed as $F=\sigma 4R_{ref}^{2}$, where ${\left[.\right]}_{+}$ = max{.,0} (notice that since σ(x) and ε(x) are assumed to be positive for compressive stress and strain, the above equation is valid for positive values of the expression in the parentheses).

### 2.3. Comparison of the Particle Mechanics Approach and the Conventional Continuum Mechanics Approach

According to the Hertz theory [43], the collinear contact force between the compressed elastic particles is a nonlinear function of the overlap, which is formed under the effect of the external load acting on the particles. For the case of thermally-assisted compaction of a granular system, such dependency is altered under the effect of an applied thermal gradient. To illustrate this influence, we work on the analytical solutions proposed in PMA and CMA for a system of stainless steel spherical particles. 304 stainless steel has the following bulk properties: E=193 GPa, k=15W/mK, ν=0.29 and $\alpha =17.3\;10^{-6}\;1/K$. The packing fraction is taken as $\phi_{s}=\pi /6\left(1-\varepsilon \right)$, and the coordination number as Z=6.

**Figure 2:**
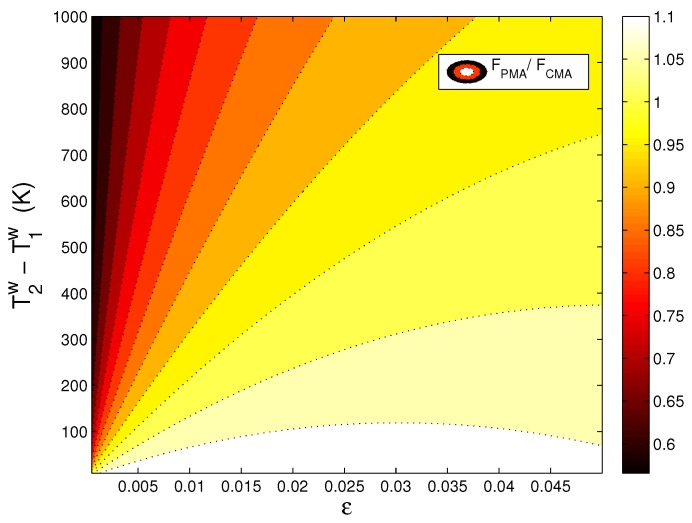
Comparison of compaction force calculated in PMA and CMA under varying thermal and mechanical loading conditions.

**Figure 3:**
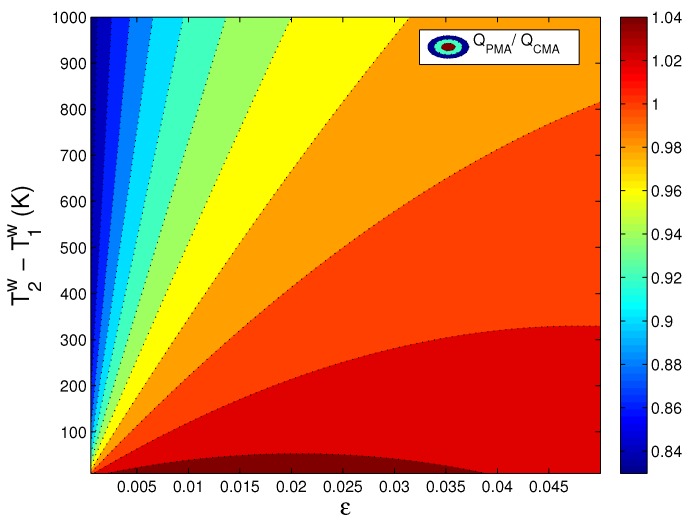
Comparison of heat transfer calculated in PMA and CMA under varying thermal and mechanical loading conditions.

In Figure 2 and Figure 3, we aim to compare the analytical solutions generated in the particle mechanics approach and in the conventional continuum mechanics approach under varying boundary conditions for the relevant thermally-assisted compaction simulation. The compaction force and the heat transferred that are calculated by these two approaches are shown in the ratio. Figure 2 and Figure 3 reflect a discrepancy between PMA and CMA particularly for the deformation range of high thermal load and low mechanical load.

## 3. Derivation of the Effective Thermal Expansion Coefficient for Thermally-Assisted Compaction of Granular Beds

Similar in spirit to earlier studies, the multi-scale approach discussed in this work is used to link the particle level information to the continuum level description of the thermally-assisted compaction process. We present a methodology to derive an effective thermal expansion coefficient for confined granular systems. The superscript mn is used to refer to the particle interactions at the contact of individual particles *m* and *n*. In this section, the quantities defined at the particle-level description and the continuum-scale description are denoted slightly different. For instance, stress and heat flux are defined as $\sigma^{mn}$ and $q^{mn}$ in PMA and $\sigma (x^{mn})$ and $q(x^{mn})$ in CMA.

Considering the case of an infinite chain of identical particles, we assume that: (i) the temperature difference between consecutive pairs is negligible compared to the change with respect to the reference temperature (i.e., $∥\left(T^{m}-T^{n}\right)/\left(T^{mn}-T_{ref}\right)∥\ll 1$ ); and (ii) the particles are locally subject to uniform compaction. Moreover, we consider an average porosity for the resembling systems of particle-scale and continuum scale analysis; therefore, $\phi_{s}$ for a given compaction strain ε is calculated as π/6(1−ε). Based on the above assumptions, the average stress calculated in PMA can be expressed as:

(17) $$\begin{array}{c} \sigma^{mn}=\frac{F^{mn}}{4R_{ref}^{2}}=\frac{C_{n}}{6}{\left[1+\alpha \left(T^{mn}-T_{ref}\right)\right]}^{1/2}{\left(\frac{\gamma^{mn}}{2R_{ref}}\right)}^{3/2} \end{array}$$

whereas the continuum-level approach predicts the particular compression stress as (valid for compressive stresses $\sigma (x^{mn})>0$):

(18) $$\begin{array}{cc} \sigma (x^{mn}) & =(\tilde{\lambda }+\tilde{\mu })\varepsilon (x^{mn})+\tilde{\alpha }\left(3\tilde{\lambda }+2\tilde{\mu }\right)\left(T(x^{mn})-T_{ref}\right)\\ & =\phi_{s}ZC_{n}{\left(\frac{3}{32\pi^{2}}\right)}^{1/2}{\left[\varepsilon \left(x^{mn}\right)\frac{3}{5}+\tilde{\alpha }\left(\frac{T\left(x^{mn}\right)+T_{ref}}{2}-T_{ref}\right)\right]}_{+}^{3/2} \end{array}$$

where $\tilde{\alpha }$ is the effective thermal expansion coefficient. Next, we enforce stress and heat flux expressions in both descriptions to be equal, that is $\sigma \left(x^{mn}\right)=\sigma^{mn}$ and $q\left(x^{mn}\right)=q^{mn}$. $x^{mn}$, $T(x^{mn})$ and $\varepsilon (x^{mn})$ are:

(19) $$\begin{array}{ccc} x^{mn} & = & \frac{x^{m}+x^{n}}{2} \end{array}$$

(20) $$\begin{array}{ccc} T(x^{mn}) & = & \frac{T^{m}+T^{n}}{2} \end{array}$$

(21) $$\begin{array}{ccc} \varepsilon (x^{mn}) & = & 1-\frac{\left|\right|x^{m}-x^{n}\left|\right|}{2R_{ref}}=\frac{\gamma^{mn}}{2R_{ref}} \end{array}$$

Finally, the equivalence of Equations (17) and (18) provides the following continuum level effective thermal expansion expression that is dependent on the applied mechanical and thermal load, σ and $T-T_{ref}$, as well as the bulk thermal expansion property of the solid.

(22) $$\tilde{\alpha }\left(\sigma ,T\right)=\frac{3}{5}\alpha +\frac{4\pi }{T-T_{ref}}{\left(\frac{\sigma }{C_{n}\phi_{s}Z{\left(6\pi \right)}^{1/2}}\right)}^{2/3}\left[1-{\left(\frac{1}{1+\alpha (T-T_{ref})}\right)}^{1/3}\right]$$

The first order approximation of the above expression is:

(23) $$\tilde{\alpha }\left(\sigma ,T\right)=\frac{3}{5}\alpha +\frac{4\pi }{3{\left(C_{n}\phi_{s}Z\right)}^{2/3}{\left(6\pi \right)}^{1/3}}\sigma^{2/3}\alpha \left[1-\frac{2}{3}\alpha \left(T-T_{ref}\right)\right]$$

To quantify the overall effect of this approximation, we implement both equations, Equations (22) and (23), in the continuum-scale solution, and we compare the required stress and heat flux particularly for the previously discussed deformation range. It is found that the two compared CMA solutions differ less than 1% under these conditions.

### An Application of the Proposed Effective Thermal Expansion Coefficient

Along with the effective mechanical properties and effective thermal conductivity (listed in Section 2.2), we implement the first order approximation of the proposed thermal expansion coefficient, Equation (23), to solve the described thermally-assisted compaction problem. The analytical solution is given in the following set of equations:

(24) $$\begin{array}{ccc} T(x) & = & \frac{\Delta T^{w}}{L_{i}}x+T_{1}^{w} \end{array}$$

(25) $$\begin{array}{c} u\left(x\right)=\alpha x\left[\frac{\Delta T^{w}}{2L_{i}}x+\left(T_{1}^{w}-T_{ref}\right)\right]-\frac{10\pi L_{i}}{\alpha \Delta T^{w}}{\left(\frac{\sigma }{C_{n}\phi_{s}Z{\left(6\pi \right)}^{1/2}}\right)}^{2/3}\times \\ \left\{{\left[1+\alpha \left(\frac{\Delta T^{w}}{L_{i}}x+T_{1}^{w}-T_{ref}\right)\right]}^{2/3}-{\left[(1+\alpha \left(T_{1}^{w}-T_{ref}\right)\right]}^{2/3}\right\} \end{array}$$

where $L_{i}$ is the initial length of the system, and σ is given by:

(26) $$\sigma^{2/3}=\frac{\alpha \Delta T^{w}}{10\pi }{\left(C_{n}\phi_{s}Z{\left(6\pi \right)}^{1/2}\right)}^{2/3}\frac{{\left[\alpha \left(\frac{T_{1}^{w}+T_{2}^{w}}{2}-T_{ref}\right)+\varepsilon \right]}_{+}}{{\left[1+\alpha (T_{2}^{w}-T_{ref})\right]}^{2/3}-{\left[1+\alpha (T_{1}^{w}-T_{ref})\right]}^{2/3}}$$

It is also worth noting that in the limiting case, when there is no thermal load, the derived solution for stress, Equation (26), approaches the solution given in conventional continuum mechanics approach, Equation (16).

## 4. Results and Discussion

In order to evaluate the effect of the proposed effective thermal expansion coefficient on the continuum-scale approach, we compare the three analytical solutions discussed in this study: (i) PMA; (ii) conventional CMA, where effective mechanical properties and effective thermal conductance are implemented; (iii) an improved continuum mechanics solution, where also the proposed thermal expansion coefficient is included, the Improved-CMA (also given in Equations (24)–(26)). We focus on four critical boundary conditions in detail; high/low thermal load and high/low mechanical deformation ranges.

**Figure 4:**
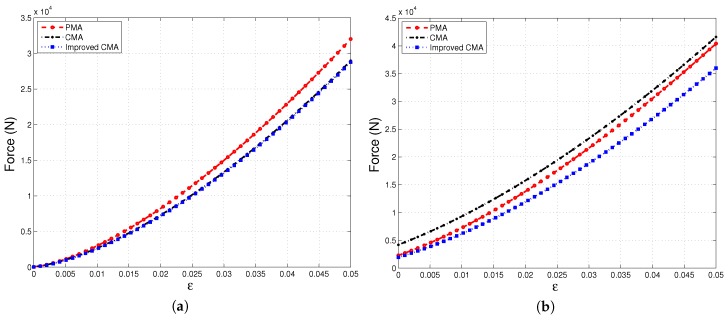
Compaction force versus strain, ε, under different thermal gradients applied at: (**a**) $T_{2}^{w}-T_{1}^{w}=40\;K$; (**b**) $T_{2}^{w}-T_{1}^{w}=1000\;K$.

In Figure 4a,b, the force needed to compress the system up to a compaction strain, ε, of 5% is traced under two different thermal load conditions. When the temperature difference between the two boundary walls is only 40K, Improved-CMA overlaps with the conventional continuum solution, as expected; whereas under high thermal load such as $T_{2}^{w}-T_{1}^{w}=1000\;K$, and particularly at low compaction strain, it is seen in Figure 4b that Improved-CMA has a better estimation of compaction force in terms of converging the PMA solution.

**Figure 5:**
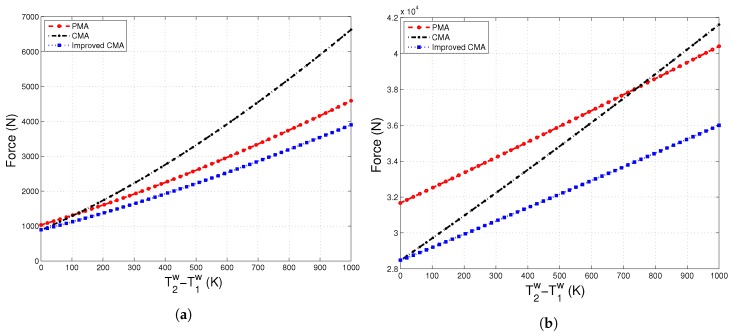
Compaction force versus thermal gradient, $T_{2}^{w}-T_{1}^{w}$, evaluated at different compaction strains: (**a**) ε=0.005; (**b**) ε=0.05.

Moreover, we evaluate the effect of thermal load on the compaction force (Figure 5a,b). Figure 5a shows that the proposed expression for the effective thermal expansion coefficient significantly improves the continuum-scale solution in predicting the compaction force. Under high mechanical load, Figure 5b suggests that Improved-CMA shows a trend in compaction force similar to the one predicted by PMA. However, there exists a difference between continuum-scale solutions and the particle-level solution at zero thermal load. The authors claim that this discrepancy stems from the calculation of the effective mechanical properties, which is also discussed in detail in the earlier study of Makse et al. [28].

**Figure 6:**
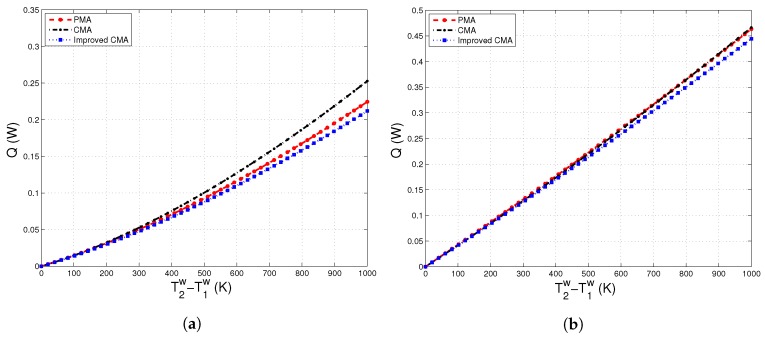
Heat flux versus thermal gradient, $T_{2}^{w}-T_{1}^{w}$, evaluated at different compaction strains: (**a**) ε=0.005; (**b**) ε=0.05.

**Figure 7:**
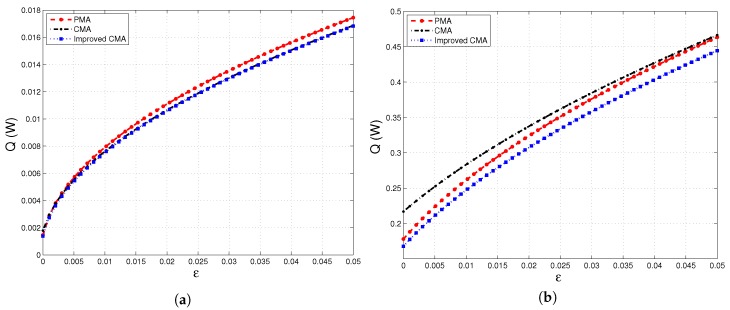
Heat flux versus compaction strain, ε, evaluated under different thermal gradients imposed at the boundary walls. (**a**) $T_{2}^{w}-T_{1}^{w}=40\;K$; (**b**) $T_{2}^{w}-T_{1}^{w}=1000\;K$.

Figure 6a,b and Figure 7a show a good agreement in terms of the heat transferred between particle-scale and continuum-scale models. Even though Figure 7b indicates that there is a difference between these two modeling approaches under varying compaction strains, it is worth noting that the maximum difference between Improved-CMA and PMA is 6%.

**Figure 8:**
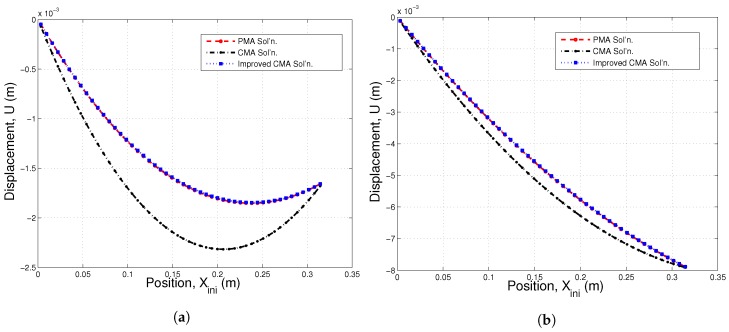
Comparison of the displacement profile of each node/particle; (**a**) $T_{2}^{w}-T_{1}^{w}=1000\;K$ and ε=0.005; (**b**) $T_{2}^{w}-T_{1}^{w}=1000\;K$ and ε=0.05.

In our previous study, we also focused on the position of individual particles under the thermally-assisted compaction process [39]. For a one-dimensional chain of particles, we noted that the relative difference in estimating the nodal position compared to the particle position between the conventional continuum mechanics approach and particle mechanics approach is up to 40% under high thermal and low mechanical load conditions. Therefore, in the current study, we plot the displacement versus the initial position of each node/particle in Figure 8a,b. It is assumed that the node in contact with the non-moving boundary is placed at x=0. We conclude that the implementation of the proposed effective thermal expansion coefficient significantly improves the conventional continuum solution in terms of predicting the displacement of the individual particle.

## 5. Conclusions

The present work centers on a multi-scale approach to bridge the gap between discrete and finite-scale solutions of a thermo-mechanically-coupled problem while introducing an effective thermal expansion coefficient. The response of a granular system under thermally-assisted compaction shows a high dependence on the thermal expansion of the particles. A discrete-system solution based on a particle mechanics approach carries out this relationship, and it successfully shows nonlinear effects on thermal strains due to thermal expansion. Despite the fact that effective medium theory enhances the conventional continuum mechanics model to a large extent, there still exists a notable discrepancy between these two approaches. In this study, we address this gap by incorporating a previously-suggested methodology to identify the effective thermal expansion property of granular materials. It is shown that the implementation of the proposed effective thermal expansion coefficient significantly improves the conventional continuum mechanics analysis, thereby resulting in better accuracy in predicting particle-level characteristics of the thermally-assisted compaction problem. The extension of the proposed approach to other multi-physics phenomena appearing in granular systems in multi-dimensional analysis is a worthwhile direction for future research.

## Figures and Tables

**Table:** 

0.49	0.49
(a)	(b)

**Table:** 

0.49	0.49
(a) H	(b) H

**Table:** 

0.46	0.46
(a) H	(b) H

**Table:** 

0.49	0.49
(a) h	(b) h

**Table:** 

0.49	0.49
(a) h	(b) h

## Abbreviations

The following abbreviations are used in this manuscript:

PMA	Particle Mechanics Approach
CMA	Continuum Mechanics Approach
**Symbol**	
$E^{m}$	Young’s modulus of the individual particle *m*
$\nu^{m}$	Poisson’s ratio of the individual particle *m*
$k^{m}$	Thermal conductivity of the individual particle *m*
$\alpha^{m}$	Thermal expansion coefficient of the individual particle *m*
$T^{m}$	Temperature of the individual particle *m*
$R^{m}$	Radius of the individual particle *m*
$a^{mn}$	Hertzian contact area between the particles *m* and *n*
$\gamma^{mn}$	Overlap at the contact of the particles *m* and *n*
$T^{w}$	Temperature at the wall surface

## References

[1] Jaeger H.M., Nagel S.R., Behringer R.P. (1996). Granular solids, liquids, and gases. Rev. Mod. Phys..

[2] Chan C.K., Tien C.L. (1973). Conductance of packed spheres in vacuum. J. Heat Transf..

[3] Kaganer M.G. (1966). Contact heat transfer in granular material under vacuum. J. Eng. Phys..

[4] Batchelor G.K., O’Brien R.W. (1977). Thermal or electrical conduction through a granular material. Proc. R. Soc. Lond. A Math. Phys. Sci..

[5] Hadley G.R. (1986). Thermal conductivity of packed metal powders. Int. J. Heat Mass Transf..

[6] Nozad I., Carbonell R.G., Whitaker S. (1985). Heat conduction in multiphase systems—II: Experimental method and results for three-phase systems. Chem. Eng. Sci..

[7] Shonnard D.R., Whitaker S. (1989). The effective thermal conductivity for a point contact porous medium: An experimental study. Int. J. Heat Mass Transf..

[8] Sridhar M.R., Yovanovich M.M. (1996). Elastoplastic contact conductance model for isotropic conforming rough surfaces and comparison with experiments. J. Heat Transf..

[9] Fletcher L.S. (1988). Recent developments in contact conductance heat transfer. Am. Soc. Mech. Eng. Trans. J. Heat Transf..

[10] Majumdar A., Tien C. (1991). Fractal network model for contact conductance. Am. Soc. Mech. Eng. Trans. J. Heat Transf. Ser. C.

[11] Bahrami M., Yovanovich M.M., Culham J.R. (2005). A compact model for spherical rough contacts. Trans. Am. Soc. Mech. Eng. J. Tribol..

[12] Majmudar T.S., Behringer R.P. (2005). Contact force measurements and stress-induced anisotropy in granular materials. Nature.

[13] Yun T.S., Santamarina J.C. (2008). Fundamental study of thermal conduction in dry soils. Granul. Matter.

[14] Chen K., Cole J., Conger C., Draskovic J., Lohr M., Klein K., Scheidemantel T., Schiffer P. (2006). Granular materials: Packing grains by thermal cycling. Nature.

[15] Chaudhuri B., Muzzio F.J., Tomassone M.S. (2006). Modeling of heat transfer in granular flow in rotating vessels. Chem. Eng. Sci..

[16] Cook C.A., Cundy V.A. (1995). Heat transfer between a rotating cylinder and a moist granular bed. Int. J. Heat Mass Transf..

[17] Natarajan V.V.R., Hunt M.L. (1998). Kinetic theory analysis of heat transfer in granular flows. Int. J. Heat Mass Transf..

[18] Li J., Mason D.J. (2000). A computational investigation of transient heat transfer in pneumatic transport of granular particles. Powder Technol..

[19] Kaneko Y., Shiojima T., Horio M. (1999). DEM simulation of fluidized beds for gas-phase olefin polymerization. Chem. Eng. Sci..

[20] Hertz H. (1881). On the contact of elastic solids. J. Reine Angew. Math..

[21] Mindlin R.D. (1949). Compliance of Elastic Bodies in Contact. J. Appl. Mech..

[22] Mindlin R.D., Deresiewicz H. (1953). Elastic spheres in contact under varying oblique forces. J. Appl. Mech..

[23] Zhu H.P., Zhou Z.Y., Yang R.Y., Yu A.B. (2007). Discrete particle simulation of particulate systems: Theoretical developments. Chem. Eng. Sci..

[24] Cundall P.A., Strack O.D.L. (1979). A discrete numerical model for granular assemblies. Geotechnique.

[25] Feng Y.T., Han K., Li C.F., Owen D.R.J. (2008). Discrete thermal element modelling of heat conduction in particle systems: Basic formulations. J. Comput. Phys..

[26] Gonzalez M., Cuitiño A.M. (2016). Microstructure evolution of compressible granular systems under large deformations. J. Mech. Phys. Solids.

[27] Vargas-Escobar W.L. (2002). Discrete Modeling of Heat Conduction in Granular Media. Ph.D. Thesis.

[28] Makse H.A., Gland N., Johnson D.L., Schwartz L.M. (1999). Why effective medium theory fails in granular materials. Phys. Rev. Lett..

[29] Makse H.A., Gland N., Johnson D.L., Schwartz L. (2001). The apparent failure of effective medium theory in granular materials. Phys. Chem. Earth Part A Solid Earth Geodesy.

[30] Zheng S., Cuitino A.M. (2002). Consolidation Behavior of Inhomogeneous Granular Beds of Ductile Particles using a Mixed Discrete-Continuum Approach. KONA Powder Part. J..

[31] Koynov A., Akseli I., Cuitiño A.M. (2011). Modeling and simulation of compact strength due to particle bonding using a hybrid discrete-continuum approach. Int. J. Pharm..

[32] Gonzalez M., Cuitiño A.M. (2012). A nonlocal contact formulation for confined granular systems. J. Mech. Phys. Solids.

[33] Walton K. (1975). The effective elastic moduli of model sediments. Geophys. J. R. Astron. Soc..

[34] Vargas W.L., McCarthy J.J. (2001). Heat conduction in granular materials. Am. Inst. Chem. Eng. J..

[35] Vargas W.L., McCarthy J.J. (2007). Thermal expansion effects and heat conduction in granular materials. Phys. Rev. E.

[36] Johnson K.L. (1987). Contact Mechanics.

[37] Lu Z., Abdou M., Ying A. (2001). 3D Micromechanical modeling of packed beds. J. Nucl. Mater..

[38] Siu W.W.M., Lee S.K. (2004). Transient temperature computation of spheres in three-dimensional random packings. Int. J. Heat Mass Transf..

[39] Küçük G., Gonzalez M., Cuitino A.M. (2016). Thermo-Mechanical Behavior of Confined Granular Systems. Lecture Notes in Applied and Computational Mechanics: Innovative Numerical Approaches for Multi-Field and Multi-Scale Problems.

[40] Markov K.Z. (2000). Elementary micromechanics of heterogeneous media. Heterogeneous Media.

[41] Norris A.N., Johnson D.L. (1997). Nonlinear elasticity of granular media. Trans. Am. Soc. Mech. Eng. J. Appl. Mech..

[42] Vargas W.L., McCarthy J.J. (2002). Stress effects on the conductivity of particulate beds. Chem. Eng. Sci..

[43] Landau L.D., Lifshitz E.M. (1959). Theory of Elasticity.

